# Regulatory mechanism of fibrosis-related genes in patients with heart failure

**DOI:** 10.3389/fgene.2022.1032572

**Published:** 2022-10-17

**Authors:** Yijing Tao, ChengJie Gao, Da Qian, Donglai Cao, Leng Han, Ling Yang

**Affiliations:** ^1^ Department of Cardiology, The Third Affiliated Hospital of Soochow University, Changzhou, China; ^2^ Department of Cardiology, Changshu Hospital Affiliated to Soochow University, Changshu No.1 People’s Hospital, Changshu, China; ^3^ Department of Geriatrics, Shanghai Jiao Tong University Affiliated Sixth People’s Hospital, Shanghai, China; ^4^ Department of Burn and Plastic Surgery-Hand Surgery, Changshu Hospital Affiliated to Soochow University, Changshu No.1 People’s Hospital, Changshu, China

**Keywords:** heart failure, hub gene, fibrosis, diagnosis, cardiac remodeling

## Abstract

**Background:** Heart failure (HF) is a complex clinical syndrome characterized by the inability to match cardiac output with metabolic needs. Research on regulatory mechanism of fibrosis-related genes in patients with HF is very limited. In order to understand the mechanism of fibrosis in the development and progression of HF, fibrosis -related hub genes in HF are screened and verified.

**Methods:** RNA sequencing data was obtained from the Gene Expression Omnibus (GEO) cohorts to identify differentially expressed genes (DEGs). Thereafter, fibrosis-related genes were obtained from the GSEA database and that associated with HF were screened out. Gene Ontology (GO) and Kyoto Encyclopedia of Genes and Genomes (KEGG) pathways analysis was carried out to analyze the biological function of fibrosis-related DEGs. The protein-protein interaction (PPI) network of hub genes was constructed *via* the STRING database. Moreover, the diagnostic value of hub genes for HF was confirmed using ROC curves and expression analysis. Finally, quantitative real time PCR was used to detect the expression levels of mRNAs.

**Results:** A total of 3, 469 DEGs were identified closely related to HF, and 1, 187 fibrosis-related DEGs were obtained and analyzed for GO and KEGG enrichment. The enrichment results of fibrosis-related DEGs were consistent with that of DEGs. A total of 10 hub genes (PPARG, KRAS, JUN, IL10, TLR4, STAT3, CXCL8, CCL2, IL6, IL1β) were selected *via* the PPI network. Receiver operating characteristic curve analysis was estimated in the test cohort, and 6 genes (PPARG, KRAS, JUN, IL10, TLR4, STAT3) with AUC more than 0.7 were identified as diagnosis genes. Moreover, miRNA-mRNA and TF-mRNA regulatory networks were constructed. Finally, quantitative real time PCR revealed these 6 genes may be used as the potential diagnostic biomarkers of HF.

**Conclusion:** In this study, 10 fibrosis-related hub genes in the HF were identified and 6 of them were demonstrated as potential diagnostic biomarkers for HF.

## 1 Introduction

Heart failure (HF) is a complex syndrome considered to be the consequence of a series of cardiovascular diseases, including coronary heart disease, valvular heart disease, cardiomyopathy, hypertension, etc. It is usually caused by a cardiac structural abnormality and/or systolic/diastolic dysfunction, which ultimately leads to a decrease in cardiac output and/or an elevation in intracardiac pressure. Its characteristic symptoms include dyspnea, orthopedic breathing, and lower limb swelling, and signs including increased jugular vein pressure and pulmonary congestion (Ponikowski et al., 2016). Population aging is currently a major demographic phenomenon and HF is extremely common among the elderly population. Approximately 24 million patients suffer from HF worldwide. Despite the new standard quadruple therapy (angiotensin receptor-neprilysin inhibitor (ARNI), β-blockers, mineralocorticoid receptor antagonists (MRAs) and sodium-glucose cotransporter 2 (SGLT2) inhibitor) and invasive therapies (revascularisation, cardiac resynchronisation therapy (CRT), implantable cardioverter defibrillators (ICDs), left ventricular assist device (LVAD) and heart transplantation) are beneficial and improve the prognosis of patients with HF (Writing Committe et al., 2021), unfortunately, the mortality and rehospitalizaion remain high and it costs patients to bear huge financial burden (Virani et al., 2020). HF is a global public health problem currently threatening human health, and has brought a heavy burden to patients’ physical and mental health and life quality.

Cardiac remodeling is typically associated with the occurrence and development of HF, and one of its most significant pathological features is myocardial fibrosis (MF). MF is thought to contribute to cardiac systolic and diastolic dysfunction and play a crucial role in elevating the risk of arrhythmia (Kong et al., 2014). Since cardiomyocytes do not have the capability to regenerate, it is mainly repaired by MF after myocardial injury. MF is defined as excessive deposition of extracellular matrix proteins in the cardiac interstitium, proliferation of cardiac fibroblasts and repairment of scar formation (Russo and Frangogiannis, 2016). MF can be quantified by late gadolinium enhancement (LGE) in cardiac magnetic resonance (CMR). A CMR study on hypertrophic cardiomyopathy showed that the magnitude of LGE progression is correlated to future implantation of ICDs, deterioration of ejection fraction, and admission for HF (Habib et al., 2021). In patients with chronic myocardial infarction scheduled for primary preventive ICD implantation, LGE identifies a subgroup with increased risk for life-threatening arrhythmias and sudden cardiac death (Boye et al., 2011). Mandawat et al. (2021) have found that the MF progression in patients with non-ischemic dilated cardiomyopathy is associated with increased hazards of all-cause mortality and heart failure-related complications.

When designing anti-fibrosis treatment strategies for patients with HF, it is essential to fully understand the mechanisms in charge of the occurrence, progression and regression of MF. For example, Eguchi et al. (2021) found that fibroblast-specific deletion of GRK5 in mice led to decreased fibrosis and cardiac hypertrophy after chronic angiotensin II infusion or after ischemic injury compared to nontransgenic littermate controls. It has been revealed that dhS1P increases collagen synthesis in cardiac fibroblasts causing fibrosis through dhS1P-JAK/STAT-TIMP1 signaling (Magaye et al., 2020). Das et al. (2018) suggested that TRAF3IP2 can mediate TWEAK/TWEAKR-induced pro-fibrotic responses in cultured cardiac fibroblasts and the heart. Moreover, Li et al. (2020) Reported that ULK1 overexpression could reverse the regulatory effect of miRNA-1297 on MF. However, due to the complexity of signaling pathways as well as the cell types involved in MF, there is a lack of effective therapies to inhibit or reverse MF nowadays (Park et al., 2019). Thus, screening for new and more MF-related markers may provide new insights into the diagnosis and treatment of HF, and this study aims to pursue potentially differentially expressed mRNAs in HF patients with MF. It is predicted that these mRNAs might be involved in the regulation of HF and MF, with high diagnostic and could become a new target for subsequent treatment.

## 2 Materials and methods

### 2.1 Data source

The gene expression profile GSE141910 on Illumina HiSeq 2500 (*Homo sapiens*) expression beadchip and GSE57338 on [HuGene-1_1-st] Affymetrix Human Gene 1.1 ST Array [transcript (gene) version] expression beadchip platform were acquired from the Gene Expression Omnibus (GEO) of NCBI (http://www.ncbi.nlm.nih.gov/gds/), respectively. Fibrosis-related genes were obtained from the GSEA database. GSE141910, comprised of left ventricular tissues 200 HF samples and 166 control samples, and GSE57338, composed of heart left ventricle tissues of 177 HF samples and 136 control samples were used as training set and external validation set, respectively.

### 2.2 Identification of differentially expressed genes

DEGs with the threshold criterion of |log_2_FC| >0.25 and adjusted *p*-value < 0.05 were screened using the limma package of the R software program (Ritchie et al., 2015). The expression heatmap and volcano plot of the DEGs were created using the “pheatmap” and “ggplot2” packages *via* R software.

### 2.3 Gene ontology and kyoto encyclopedia of genes and genomes analysis

The intersection of DEGs and fibrosis-related genes was carried out using the Venn Diagram package, and the fibrosis-related DEGs were used for subsequent analysis. The R package “ClusterProfiler” was used to implement the functional annotation of Gene ontology (GO) enrichment analysis and Kyoto Encyclopedia of Genes and Genomes (KEGG) pathway analysis of the DEGs and fibrosis-related DEGs (Yu et al., 2012a). Adjusted *p* < 0.05 was considered statistically significant.

### 2.4 Protein-protein interaction network construction

Herein, STRING was used to analyze the functional connections and interactions between proteins. Then the visualization of the PPI network was achieved based on Cytoscape (https://cytoscape.org/, version 3.7.2), with the hub genes screened by the cytoHubba plug-in of Cytoscape software.

### 2.5 MiRNA-hub gene-interaction analysis

MiRNet (https://www.mirnet.ca/), a convenient online database that mainly focuses on miRNA-target interactions was used in the current study to predict the miRNAs targeting hub genes (Fan and Xia, 2018). In order to comprehensively and accurately excavate the regulatory relationship between miRNAs and hub genes, the miRNAs of hub genes were comprehensively predicted by miRNet database. We construct the regulatory network by Cytoscape based on the prediction of mRNA-miRNA.

### 2.6 Transcription factor-hub gene-interaction analysis

NetworkAnalyst database (https://www.networkanalyst.ca/) was used to predict the TFs that could regulate HF-associated hub genes. Next, Pearson correlation analysis was implemented to screen more stringent TFs of key genes, and TFs with *p*-value<0.05 and the absolute value of correlation ≥0.4 were regarded as potential TFs of key genes. Moreover, the TFs-hub genes network was visualized by Cytoscape.

### 2.7 Receiver operating characteristic curve analysis

Then ROC curve analysis was implemented to classify the sensitivity and specificity of the hub genes for HF diagnosis. We calculated the area under the curve (AUC) using the statistical package “pROC” in R software (Robin et al., 2011). The boxplot of hub genes expression was drawn using the “ggplot2” in R package.

### 2.8 qPCR of hub genes

Finally, to investigate the roles of hub genes in HF, quantitative real time PCR (RT-qPCR) was used to detect the expression levels of mRNAs in plasma samples from HF patients (*n* = 10) and healthy controls (*n* = 10), which obtained from Changshu No.1 People's Hospital. The clinical features of these HF patients and healthy controls have shown in Table 1. Among the 10 HF patients, 4 were heart failure after myocardial infarction, 3 were ischemic cardiomyopathy, 2 were dilated cardiomyopathy and 1 was hypertrophic cardiomyopathy. Blood samples of HF patients were collected within 2 h after admission to CCU or cardiology ward. All blood samples were collected in EDTA anticoagulant tubes and stored in the central laboratory −80°C refrigerator until thawed for analysis. Plasma was isolated by a double-centrifugation protocol as previously described (Tsui et al., 2014). Total RNA from plasma samples were isolated using TRIzol cracking method. RNAs were eluted with 14 of µl RNAse-free water and stored in Low DNA binding Eppendorf tubes (Eppendorf) at−80°C. Next, total RNA was reverse transcribed into complementary DNA (cDNA) using the iScript™ cDNA Synthesis Kit (Bio-Rad, Hercules, CA, United States) based on the manufacturer’s procedure. Moreover, quantitative real time PCR was performed using SYBR Green Premix Ex Taq™ (Takara, Japan) and the Applied Biosystems 7500 Real-time PCR System (Applied Biosystems, Inc., Carlsbad, CA, United States). Finally, the relative expression level of each lncRNA was calculated using the 2^−ΔΔCt^ method, ΔΔCt = (CtRNA − Ctβ-actin) BC cells − (CtRNA − Ctβ-actin) normal cells, and fold change = 2^−ΔΔCt^. Primer sequences and annealing temperatures of quantitative real time PCR could be found in Table 2.

**TABLE 1:** The clinical characteristics of HF group and control group.

Parameter	HF group (N = 10)	Control group (N = 10)	*p* value
Age, years	77.30 ± 6.45	67.70 ± 10.07	0.152
Gender (Male/Female)	9/1	8/2	0.531
BMI, kg/m^2^	22.16 ± 2.80	25.01 ± 2.87	0.982
LVEF, %	41.80 ± 12.69	25.01 ± 2.87	0.005
LVEDD, mm	56.70 ± 10.54	44.60 ± 4.38	0.003
BNP, pg/ml	609.00 (278.75,1103.00)	59.50 (30.75,83.00)	<0.001
CRP, mg/l	10.85 ± 9.25	0.95 ± 0.95	0.008

BMI, body mass index; LVEF, left ventricular ejection fraction; LVEDD, left ventricular end-diastolic dimension.

**TABLE 2:** Primers used in Quantitative PCR.

Primers	Sequence (5’→3′)	
IL10	Forward	5′-ATT​CAC​CTT​CCA​GTG​TCT​CGG-3′
	Reverse	5′-GAC​CTC​AAG​TGA​TCC​ACC​CG-3′
JUN	Forward	5′-CTC​AGA​CAG​TGC​CCG​AGA​TG-3′
	Reverse	5′-TGT​GCC​ACC​TGT​TCC​CTG​AG-3′
KRAS	Forward	5′-CTG​CTG​CTG​TGG​ATA​TCT​CCA-3′
	Reverse	5′-ATG​TTC​AAA​GCA​TCA​GCC​ACC-3′
PPARG	Forward	5′-CAC​TAC​TGT​TGA​CTT​CTC​CAG​CAT​T-3′
	Reverse	5′-CAT​GAG​GGA​GTT​GGA​AGG​CT-3′
STAT3	Forward	5′-AGG​CAT​GTC​TCC​TTG​CGT​GT-3′
	Reverse	5′-ATG​AAC​TGA​ATG​AAG​ACG​CCA-3′
TLR4	Forward	5′-CAA​ACG​GCT​GCT​GAG​GGT-3′
	Reverse	5′-AAT​CTG​GAT​GAT​GAA​GTT​ACA​CCT​C-3′
GAPDH	Forward	5′-GTG​AAG​CAG​GCG​TCG​GA-3′
	Reverse	5′-CTC​TCT​TCC​TCT​TGT​GCT​CTT​GC-3′

## 3 Results

### 3.1 Identification of differentially expressed genes in heart failure and functional enrichment analysis

**FIGURE 1:**
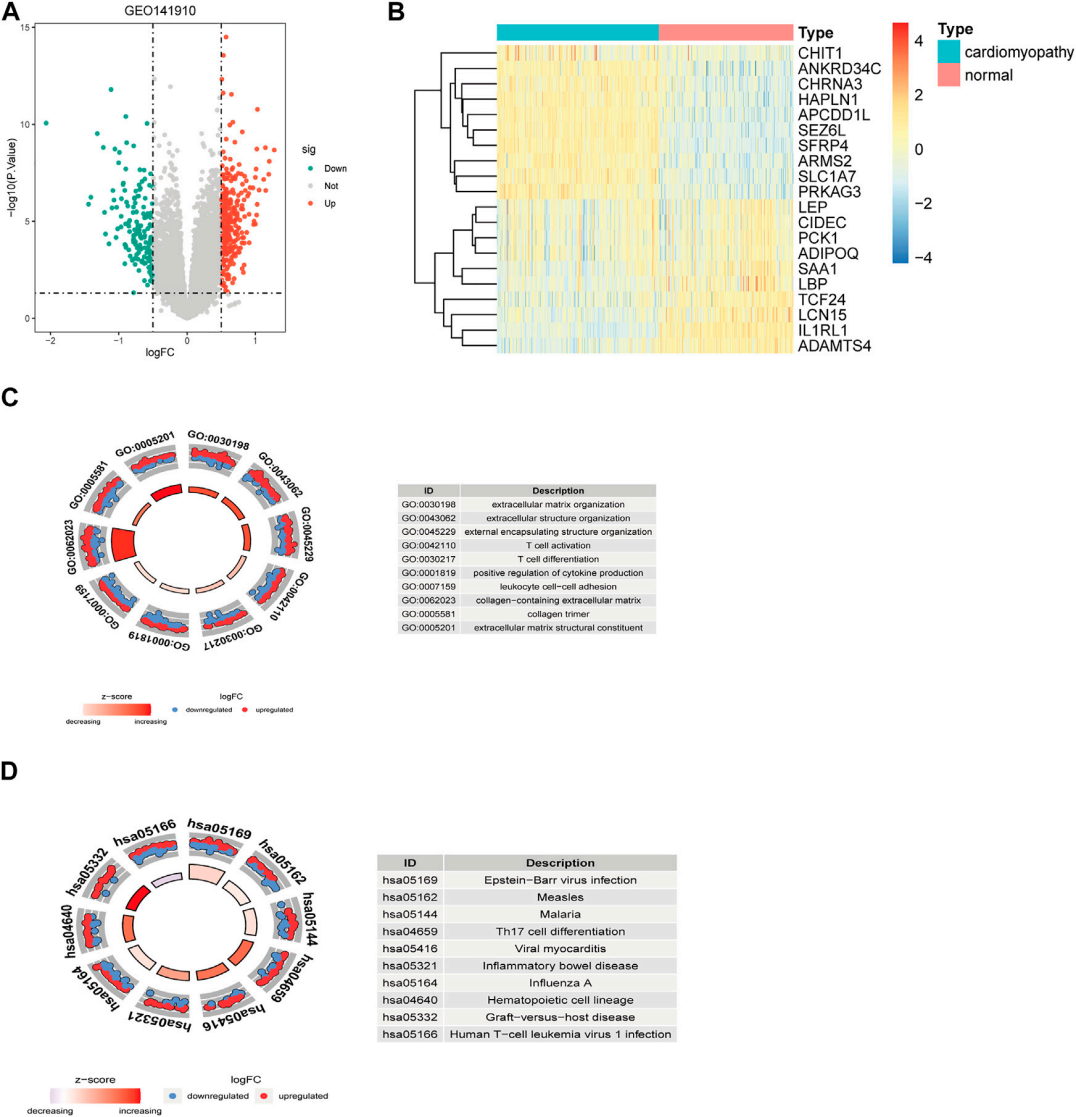
Identification and functional enrichment analysis of DEGs between the HF samples and control samples in GSE141910 datasets. Volcano plot **(A)** and heatmap **(B)** of DEGs. GO enrichment **(C)** and KEGG pathway enrichment results **(D)** of the DEGs. Green, downregulated; red, upregulated; grey, not differential expressed. DEGs, differentially expressed genes.

Using GSE141910, a total of 3,469 DEGs were identified between 200 heart failure samples and 166 healthy samples (Figures 1A,B, Supplementary Table S1), among which 2,052 genes were significantly upregulated, and 1,417 genes were significantly downregulated in HF patients compared with healthy samples. These DEGs were significantly enriched into 375 BPs, 15 CCs, 19 MFs, 328 KEGGs (Supplementary Tables S2, S3). As shown in Figure 1C, the GO analysis results for the DEGs indicated these DEGs were mainly involved in extracellular matrix organization, extracellular structure organization in the biological process category, collagen-containing extracellular matrix in the cellular component category, and extracellular matrix structural constituent in the molecular function category. Moreover, KEGG pathway analysis revealed that these DEGs were mainly enriched in pstein-barr virus infection, human T-cell leukemia virus 1 infection, influenza A (Figure 1D).

### 3.2 Extraction the fibrosis-related differentially expressed genes and construction of the protein-protein interaction network

**FIGURE 2:**
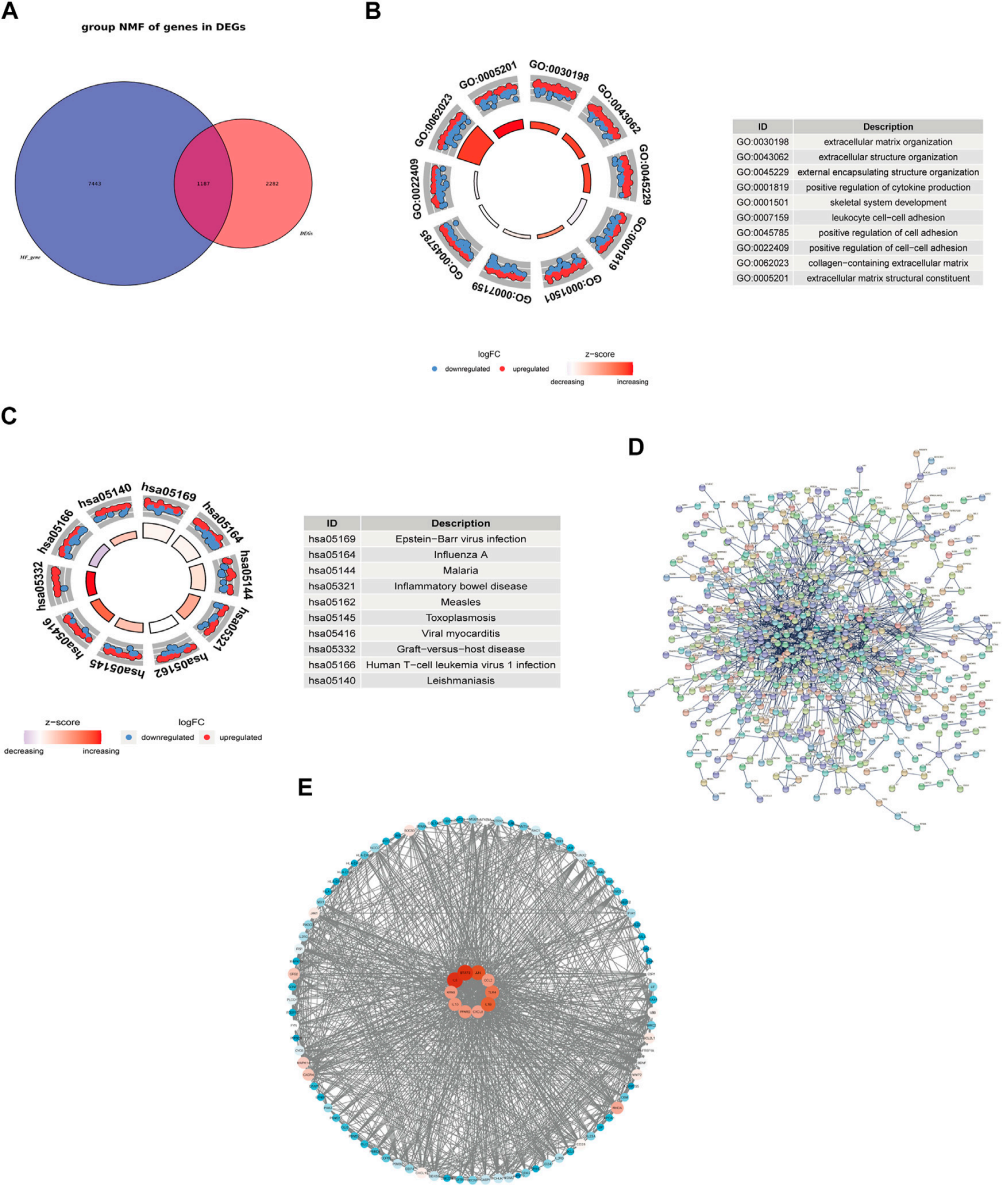
Functional enrichment analysis and exploring the interaction of fibrosis-related DEGs. Venn Diagram showed the intersection of DEGs and fibrosis-related genes **(A)**. GO enrichment **(B)** and KEGG pathway enrichment results **(C)**. PPI network of the interaction of fibrosis-related DEGs **(D)**. PPI network of the top 100 genes and the top 10 genes with the highest degrees **(E)**.

A total of 8630 fibrosis-related genes were collected in the genecard database. After intersecting 3469 DEGs and 8630 fibrosis-related genes, a total of 1187 fibrosis-related DEGs were obtained (Figure 2A). Functional enrichment analysis showed that these fibrosis-related DEGs were significantly enriched into 1651 BPs, 88 CCs, 72 MFs, 317 KEGGs (Supplementary Tables S4, S5). As shown in Figure 2B, the GO analysis indicated that fibrosis-related DEGs were mainly associated with extracellular matrix organization, extracellular structure organization in the biological process category, collagen-containing extracellular matrix in the cellular component category, and extracellular matrix structural constituent in the molecular function category. In addition, KEGG pathway analysis revealed that fibrosis-related DEGs were mainly enriched in epstein-barr virus infection, human T-cell leukemia virus 1 infection, influenza A, which was consistent with the enrichment results of DEGs (Figure 2C).

To further explore the protein interaction of fibrosis-related DEGs, we used the STRING database to construct a PPI network (Figure 2D). This network has a total of 1145 nodes and 1583 edges. PPI network of the top 100 genes was shown in Figure 2E and the top 10 genes with the highest degrees were selected and defined as hub genes in HF, including KRAS, JUN, IL6, IL1β, IL10, CXCL8, CCL2, TLR4, STAT3, and PPARG. The degrees of the node were correlated with the tint of the color, from the blue to the red.

### 3.3 The ROC curve analysis and expression analysis of hub genes in train set and validation set

**FIGURE 3:**
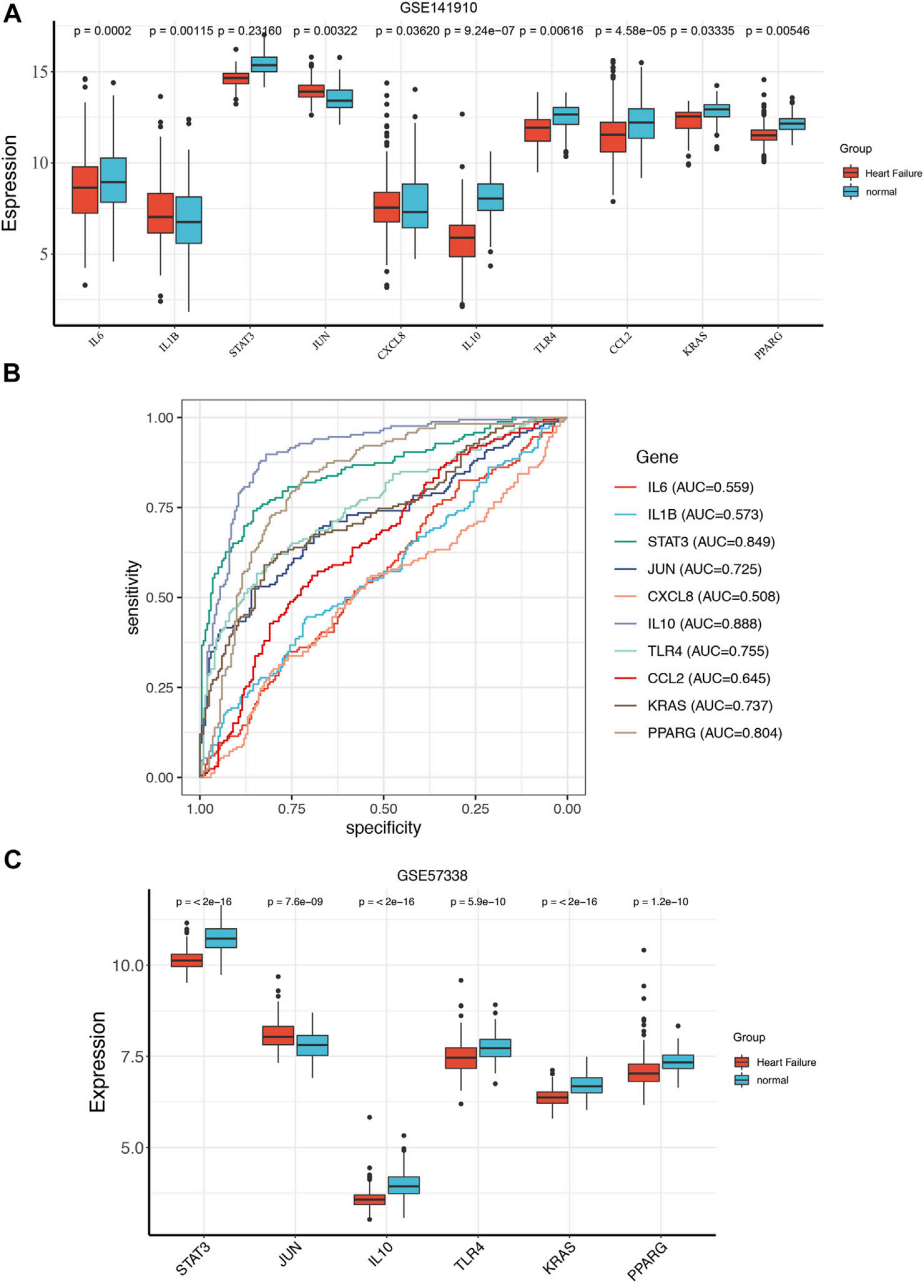
The ROC curve analysis and expression analysis of hub genes in train set and validation set. The expression of the hub genes between the HF and normal group in GSE141910 **(A)**. ROC curve evaluated the diagnostic value of hub genes for HF in GSE141910 **(B)**. The expression of the hub genes between the HF and normal group in GSE57338 **(C)**.

To get more robust key fibrosis-related genes in HF, we firstly observed the expression levels of hub genes between the HF and healthy samples in GSE141910. Interestingly, we found that the expressions of IL6, KRAS, CCL2, IL10, TLR4, STAT3 and PPARG in HF patients were down-regulated compared with healthy samples, while the expressions of JUN, IL1β and CXCL8 were up-regulated in HF samples compared with healthy samples (Figure 3A). Moreover, ROC curves showed the except for IL6, IL1β, CXCL8 and CCL2, whose AUC values were 0.559, 0.573, 0.508 and 0.645, the AUC of all other genes were greater than 0.7 (Figure 3B), indicating that IL10, JUN, KRAS, PPARG, STAT3 and TLR4 might be used as biomarkers for distinguishing HF and non-HF samples. The thresholds of IL10, JUN, KRAS, PPARG, STAT3, and TLR4 were 6.91, 13.44, 12.84, 11.75, 15.1, and 12.5, respectively. The included individuals were assigned into low- and high-expressed group by the threshold of each mark gene, which was presented in Table 3. Furthermore, we also examined the expressions of IL10, JUN, KRAS, PPARG, STAT3 and TLR4in external GSE57338 dataset. Excitingly, the expressions of KRAS, IL10, TLR4, STAT3 and PPARG in HF patients were significantly down-regulated, the expression of JUN was significantly up-regulated in HF patients compared with healthy samples (Figure 3C), which was consistent with the result of GSE141910. After that, we examined the expressions of 6 hub genes in GSE5406, GSE42955 and GSE116250 and the results showed the same trend of gene expression in these datasets compared with our verification results (Supplementary Figure S1). Thus, IL10, JUN, KRAS, PPARG, STAT3 and TLR4 might play key roles in HF, and were defined as the ultimately hub genes.

**TABLE 3:** The distribution of HF and normal individuals between the low- and high-expression group.

Gene	Low-expression		High-expression		*p* value
	HF	Normal	HF	Normal	
IL10	164	18	36	148	<0.001
JUN	30	87	170	79	<0.001
KRAS	165	69	35	97	<0.001
PPARG	141	26	59	140	<0.001
STAT3	172	48	28	118	<0.001
TLR4	160	63	40	103	<0.001

### 3.4 The potential regulatory mechanisms of ultimately hub genes

**FIGURE 4:**
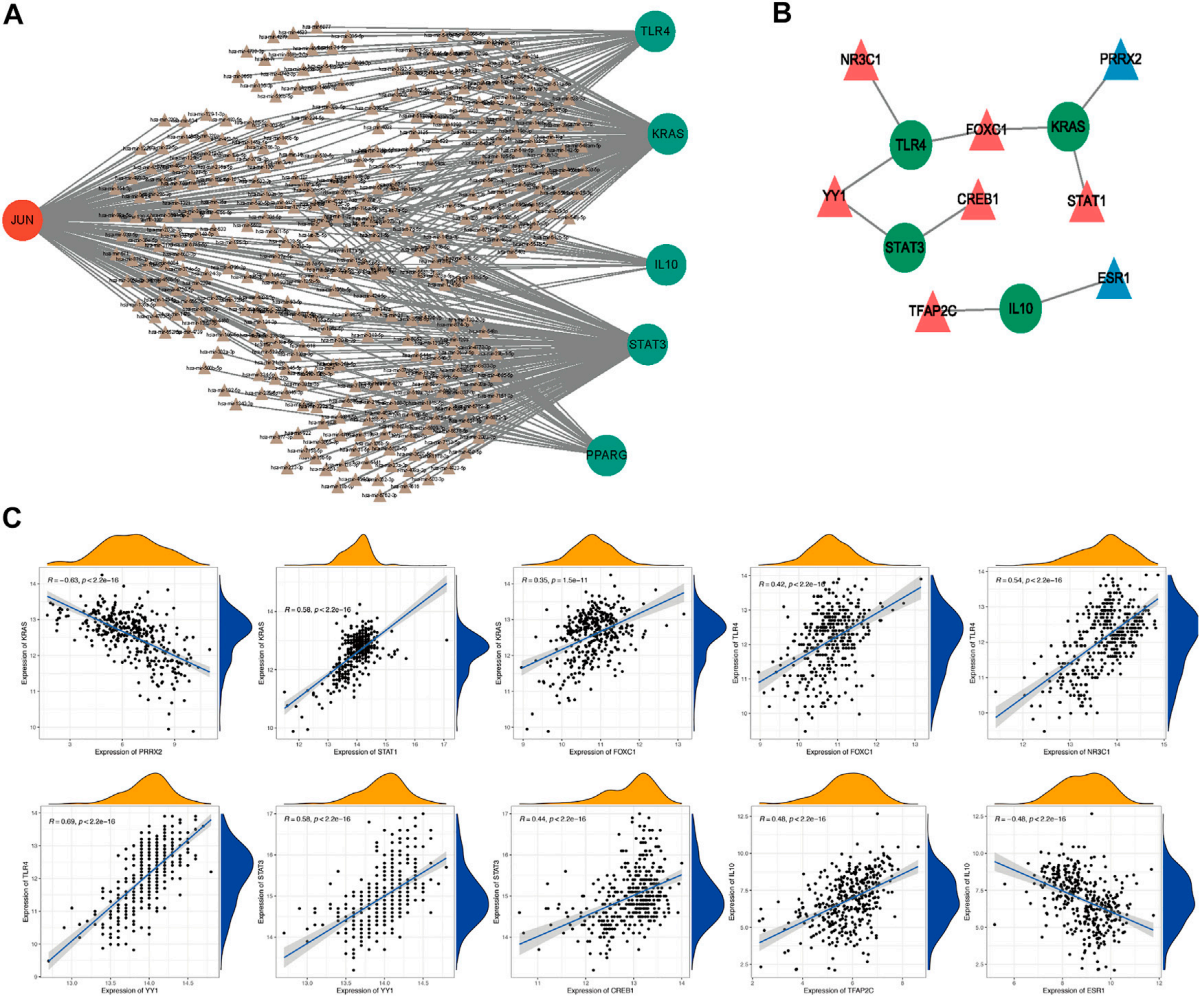
The potential regulatory mechanisms of ultimately hub genes. The regulatory relationships between the target genes and their miRNAs **(A)**. Green, downregulated; red, upregulated; Circle, hub gene; Triangle, miRNA. The interaction network consists of 4 hub genes and 8 TFs **(B)**. Blue, downregulated; red, upregulated; Circle, hub genes; Triangle, Transcription Factor. The correlation analysis of 4 hub genes and their potential TFs **(C)**.

To further explore the potential regulatory mechanisms of ultimately hub genes, we firstly predicted potentially regulating miRNAs of ultimately hub genes. The regulatory relationships between the ultimately hub genes and their potentially regulating miRNAs were established using Cytoscape software. As shown in Figure 4A, we found that 148 miRNAs (ie, hsa-miR-17-5p) might regulate the expression of STAT3, 144 miRNAs (ie, hsa-miR-15a-5p) might regulate the expression of JUN, 132miRNAs (ie, hsa-miR-16-5p) might regulate the expression of KRAS, 43 miRNAs (ie, hsa-miR-1-3p) might regulate the expression of TLR4, 34 miRNAs (ie, hsa-miR-215-5p) might regulate the expression of PPARG, 19 miRNAs (ie, hsa-miR-194-5p) might regulate the expression of IL10.

Moreover, we also investigated the potential regulatory TFs of ultimately hub genes, and the interaction network consisting of 8 TFs and 4 ultimately hub genes was constructed. As illustrated in Figure 4B, we found that NR3C1 might be a positively related TF of TLR4, YY1 might be a positively related TF of TLR4 and STAT3, CREB1 might be a positively related TF of STAT3, FOXC1 might be a positively related TF of TLR4 and KRAS, STAT1 might be a positively related TF of KRAS, TFAP2C might be a positively related TF of IL 10, and ESR1 might be a negatively related TF of IL10, PRRX2 might be a negatively related TF of KRAS. Therefore, we speculated that NR3C1, YY1, FOXC1, CREB1, STAT1, TFAP2C, ESR1, and PRRX2 might affected HF progression by regulating TLR4, STAT3, KRAS, and IL10 and the correlation analysis of 4 hub genes and their potential TFs was shown in Figure 4C.

### 3.5 Validation of the mRNA expression of ultimately hub genes between heart failure and healthy samples by RT-qPCR

**FIGURE 5:**
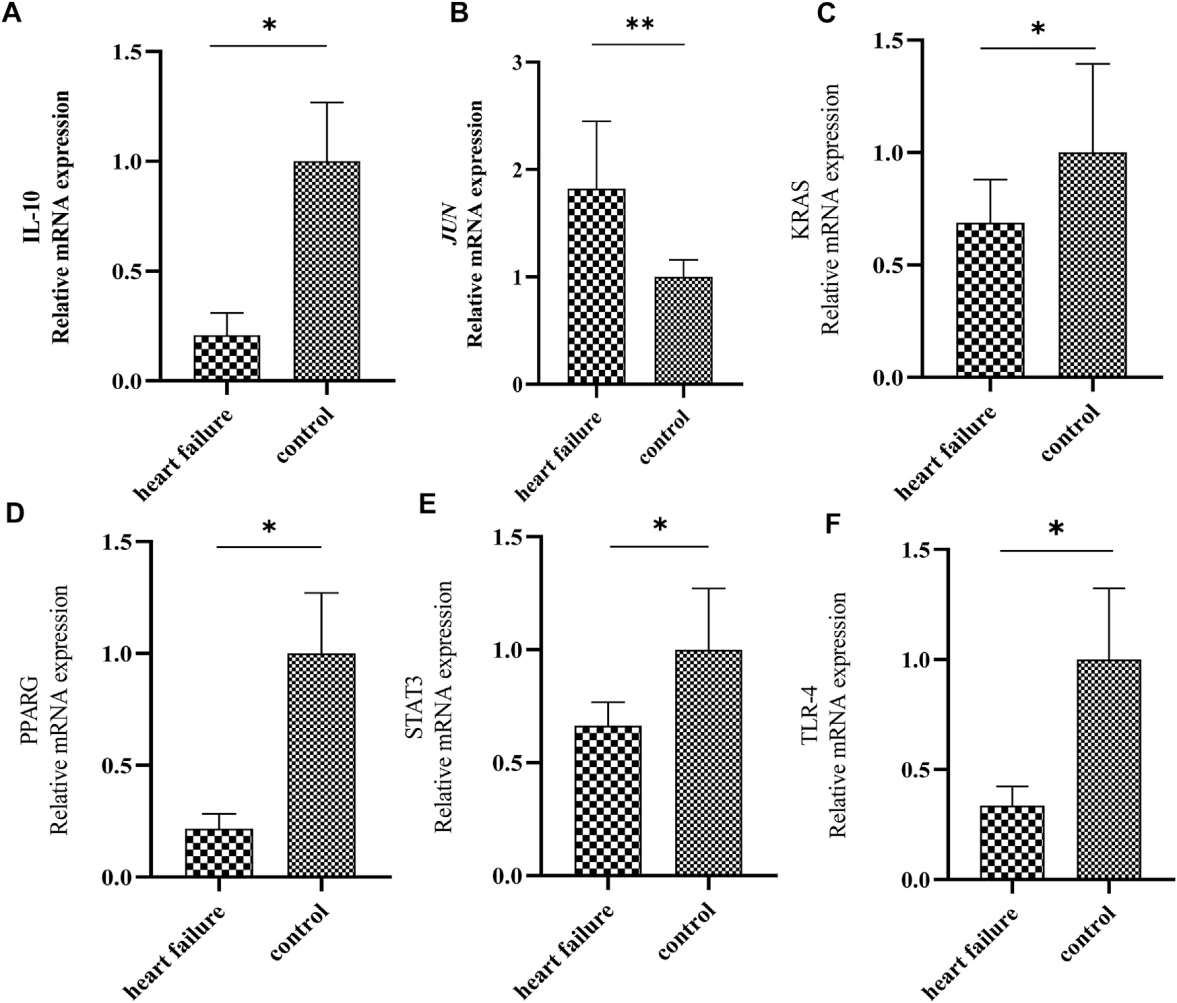
Validation of the expression of 6 hub genes by quantitative real time PCR. The levels of IL-10 **(A)**, JUN **(B)**, KRAS **(C)**, PPARG **(D)**, STAT3 **(E)** and TLR4 **(F)** in plasma samples from patients with HF and healthy controls were measured by qPCR. Results were shown as mean ± SD. * *p* < 0.05 ** *p* < 0.01 vs. control. GAPDH was used as housekeeping gene.

At last, we determined the expression levels of IL10, JUN, KRAS, PPARG, STAT3 and TLR4 between HF and healthy samples by RT-qPCR. Notably, we found that the expression of JUN was significantly elevated, and the expressions of PPARG, KRAS, IL10, TLR4 and STAT3 were significantly down-regulated in HF plasma samples compared to normal controls (Figure 5). Those results were consistent with the RNA sequencing results in GSE141910 and GSE57338.

## 4 Discussion

HF is a complex clinical syndrome with severe morbidity, mortality, and rehospitalization rates worldwide requiring long-term treatment management, which imposes a burden on patients’ health and economy (Gerber et al., 2015). MF is one of the typical pathological features of end-stage HF, and it is a strong determinant of poor prognosis as well, predicting sudden cardiac death and ventricular tachycardia independently (Assomull et al., 2006). Due to the complexity of its mechanism, early identification, timely inhibition and reversal of MF remain to be studied. The effective targets to be developed and their corresponding therapeutic drugs are currently the research focuses and have extensive prospects in the future. Hence, we explored the fibrosis-related biomarkers in HF to provide a theoretical basis for understanding disease mechanisms and clinical diagnosis, which may be therapeutic target of HF.

Based on this study, the fibrosis-related DEGs between HF patients and normal control groups were found. Then, the PPI network of these differentially expressed fibrosis-related genes was constructed. According to the result of ROC, 6 hub genes (PPARG, KRAS, JUN, IL10, TLR4, STAT3) related to fibrosis with high specificity and sensitivity used to diagnose HF were determined as biomarkers ultimately. Based on the fact that myocardial tissue is difficult to obtain, which is contrary to tumor tissue, we conducted RT-qPCR to detect the expression levels of 6 hub genes in HF patients. The results were consistent with the RNA sequencing results in GSE141910 and GSE57338. Liquid biopsy is a powerful technique that could non-invasive detect biomarkers and monitors disease progression by collecting non solid biological tissues, such as blood samples. At present, this technology is widely used in tumor screening and early diagnosis (Chen and Zhao, 2019), and it is also promising in the cardiovascular field (Bayes-Genis and Lanfear, 2019). This result also revealed the potential value and prospect of liquid biopsy in the early diagnosis and progress monitoring of HF. In addition, we have noticed that epigenetic changes in cell-free DNA (cfDNA) are widespread in human diseases, including 5-methylcytosine (5 mC), 5-hydroxymethylcytosine (5hmC) and nucleo-some positioning (NP) (Yu et al., 2020). Due to the abundant genetic and epigenetic information carried in cfDNA, it can be detected by liquid biopsy and may revolutionize the traditional screening and treatment of various human disorders (Diaz and Bardelli, 2014).

In our interaction network, there are totally consisting of 8 TFs and 4 ultimately hub genes and we found that TLR4, STAT3, KRAS, and IL10 might be regulated by NR3C1, YY1, FOXC1, CREB1, STAT1, TFAP2C, ESR1, and PRRX2. TLR4, as one of the Toll-like receptors, is a member of the interleukin-1 receptor family and is an important regulator of inflammation. Activation of TLR4 leads to the progression of cardiac hypertrophy and injury (Katare et al., 2020). Liu et al. (2015) concluded that the expression and pro-inflammatory function of TLR4 are up-regulated after myocardial infarction, which exacerbates HF in rats. Zhang et al. (2019) found FOXC1 up-regulates the expression of toll-like receptors in myocardial ischemia. In addition, it has been revealed that NR3C1 can affect the expression of TLR4 while the research on interaction between YY1 and TLR4 was limited (van Dokkum et al., 2022). STAT3, a transcription factor, plays a protective role in the cardiovascular diseases and the deletion of STAT3 in cardiomyocytes makes the heart more vulnerable to chronic pathological lesion (Kurdi et al., 2018). Animal research data by Deshpande et al. (2018) showed that activation of STAT3 has a protective effect on acute HF. YY1 also has been found to be an activator of STAT3 while interaction between STAT3 and CREB1 is not clear (Tsui et al., 2014; Chen et al., 2019). KRAS is one of the most common oncogenes in human beings and has been widely reported in tumor-related studies in the past decades, but is very limited in the cardiovascular field. Fish et al. (2020) demonstrated that active KRAS expression in the endothelium is sufficient to cause vascular malformations. KRAS gene mutations in Noonan syndrome have been reported to be associated with a high incidence of congenital heart defects (Pierpont and Digilio, 2018). KRAS gene mutation is associated with HF, and there is a lack of research on gene deletion and activation. Our results show that KRAS is down-regulated in HF, which has potential diagnostic value and may be regulated by FOXC1, PPRX2 and STAT1, but the potential mechanism is still unclear. IL-10 is an anti-inflammatory cytokine and regulates inflammatory responses of mononuclear phagocytes. Studies have shown that IL-10 exerts its protective effect through its anti-inflammatory activity. In patients with metabolic syndrome, a higher level of IL-10 is associated with a lower incidence of coronary artery disease (Barcelos et al., 2019). Kaur et al. found in the rat model that membrane-bound IL-10 protein and mRNA levels decreased 4, 8, and 16 weeks after myocardial infarction, which illustrates the relationship between the decrease in IL-10 and the decline in cardiac function (Cuadros et al., 2006). However, there are few studies between IL10 and TFAP2C or ESR1 at present and further experimental verification is required.

The other two hub gene are PPARG (PPARγ) and JUN (c-JUN), which are out of interactive network. PPARG is a member of the peroxisome proliferator-activated receptor family, which is enriched in the adipose tissue and extra-adipose tissues, such as the heart and the vascular wall. Legchenko et al. (2018) reported that deletion of PPARG in cardiomyocytes brings about biventricular systolic dysfunction as well as intramyocellular lipid accumulation in animal models. And PPARG agonists were proven to have the ability to recover heart function in animal models of HF after myocardial infarction (Yu et al., 2012b). JUN is a member of the AP-1 transcription factor family and participates in the development of the embryonic heart (Eferl et al., 1999). JUN N-terminal kinase (JNK) plays an important role in myocardial hypertrophy and cardiac ischemia/reperfusion injury (Shvedova et al., 2018). Petrich et al. (2004) have revealed a marked stiffening of JNK-activated animal hearts, mainly associated with the remodeling of specific extracellular matrix components. Another animal study showed that inhibiting JUN signaling prevents cardiac hypertrophy (Sundaresan et al., 2012). The expression of these two hub genes is consistent with the trend of our results, PPARG was down-regulated while JUN was up-regulated in HF, which may be key genes and therapeutic target in HF. In summary, the TF-mediated network may be vital for HF development, the genes involved in the network might have the promising potential for HF diagnosis and therapy.

Increasing evidence has suggested that multi-omics driven discoveries and incorporation of additional clinical features may be more helpful in the clinical diagnosis and treatment of HF (Zhang et al., 2020; Tayanloo-Beik et al., 2021; Wu et al., 2021). Unfortunately, the current lack of multi-omics data in public databases and the very few available clinical data limit the analysis. Therefore, further exploration of more accurate markers based on multi-omics data and clinical information is necessary. Notably, we found that the expression of JUN was significantly elevated, and the expressions of PPARG, KRAS, IL10, TLR4 and STAT3 were significantly down-regulated in HF samples compared to normal controls in GSE141910, GSE57338 and our clinical samples. Thus, we speculated that PPARG, KRAS, IL10, TLR4 and STAT3 might play key roles in the clinical diagnosis and treatment of HF. In addition, a growing number of studies have suggested that combining multi-omics data may be more useful for clinical diagnosis (Yu et al., 2019; Haas et al., 2021; Wu et al., 2021). For example, Hass et al. showed a possible use of distinct molecules like succinic acid as an (early) biomarker and interventional target in HF through using multi-omics data (Haas et al., 2021). Specifically, methylation variation associated with the development of aortic atheroma is detectable in peripheral blood leucocytes prior to the development of vascular lesions (Lund et al., 2004). Different patterns of DNA methylation in peripheral blood are associated with risk of ischemic heart disease and coronary events (Baccarelli et al., 2010; Error in End Matter, 2018). Hence, we will further focus on the methylation levels of KRAS, IL10, TLR4 and STAT3, and further determine the risk of heart failure and patient stratification by the combination of methylation and transcriptional expression in the future.

In conclusion, we conducted an integrated analysis using both bioinformatics data and literature-based knowledge database to explore the hub genes of MF in HF. The miRNet database and NetworkAnalyst database were used to construct and analyze the target gene-miRNA regulatory network and target gene-TF regulatory network of 6 hub genes. In this study, we identified 6 characteristic genes related to fibrosis, and further explored that these biomarkers may provide new diagnostic and therapeutic targets for HF patients and provide new insights into the pathogenesis of MF in HF patients. Next, we will expand the sample size and further reveal the potential mechanisms of these 6 hub genes through *in vitro* and *in vivo* experiments.

## Data Availability

The datasets presented in this study can be found in online repositories. The names of the repository/repositories and accession number(s) can be found in the article/Supplementary Material

## Supplementary material

Supplementary Table S1 The gene expression profile GSE141910.

Supplementary Table S2 The DEGs were enriched into 375 BPs, 15 CCs and 19 MFs.

Supplementary Table S3 The DEGs were enriched into 328 KEGGs.

Supplementary Table S4 The fibrosis-related DEGs were enriched into 1651 BPs, 88 CCs and 72 MFs.

Supplementary Table S5 The fibrosis-related DEGs were enriched into 317 KEGGs.

Supplementary Figure S1 Validation of the expression of 6 hub genes in GSE5406, GSE42955 and GSE116250.
