# Supplementary figures and images for: Regulatory mechanism of fibrosis-related genes in patients with heart failure
Source: Front Genet. 2022 Oct 17;13:1032572. doi: 10.3389/fgene.2022.1032572 (PMC9618712; doi:10.3389/fgene.2022.1032572)

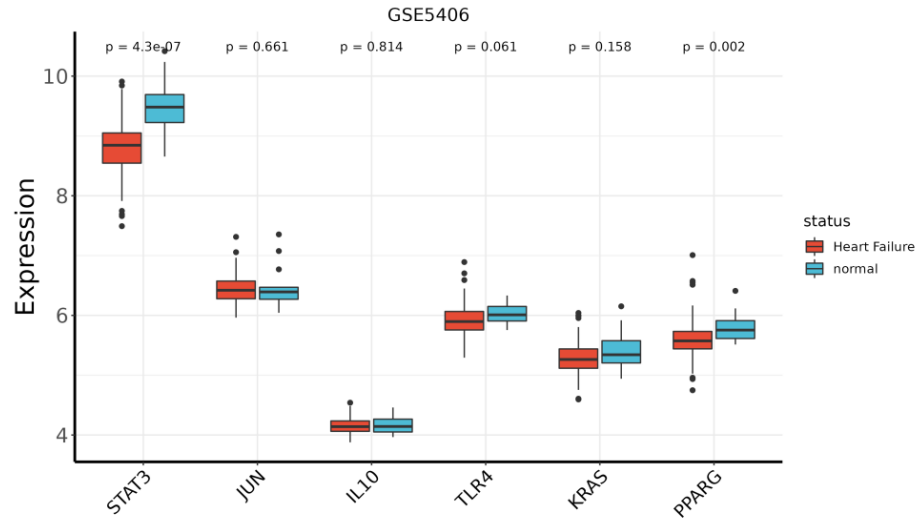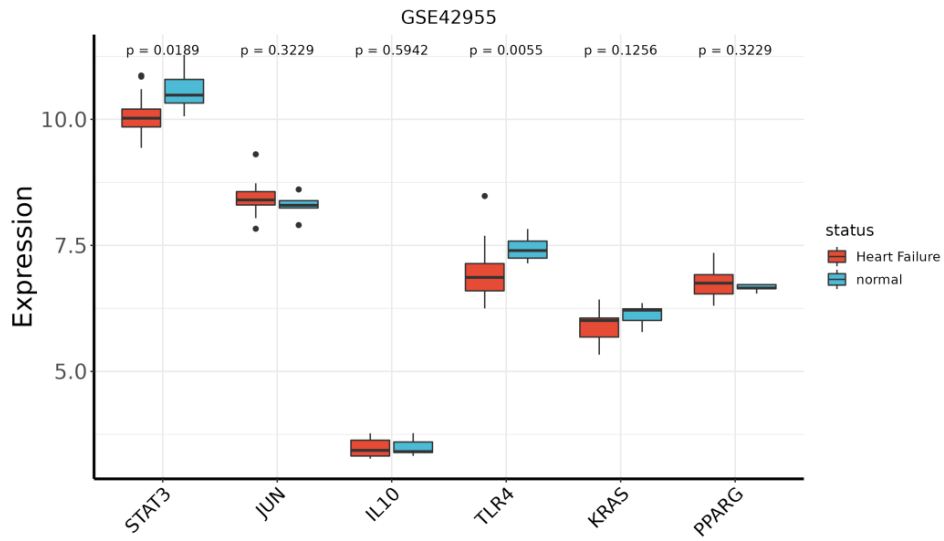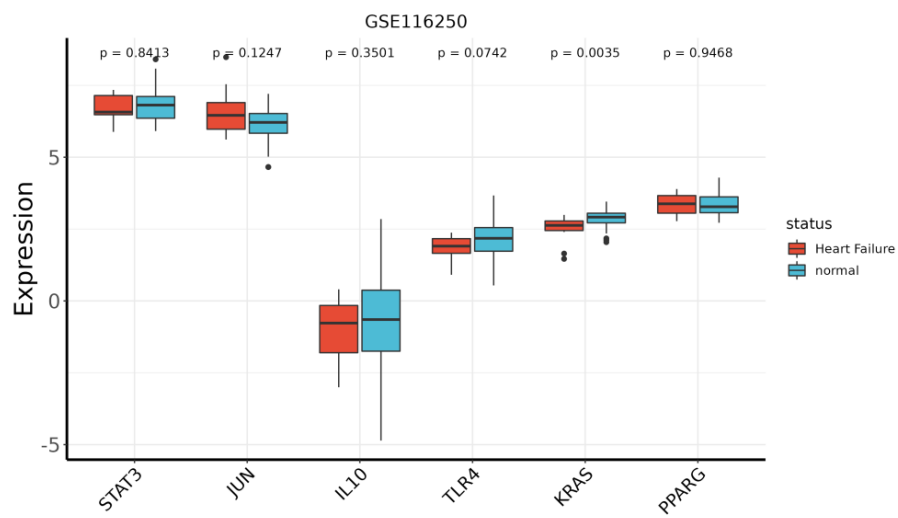

Supplement: Supplementary file 3 [file Presentation1.PDF]
